# Economic Evaluation Protocol and Statistical Analysis Plan for the Cost-Effectiveness of a Novel Australian Stroke Telemedicine Program; the Victorian Stroke Telemedicine (VST) program

**DOI:** 10.3389/fneur.2020.602044

**Published:** 2021-01-21

**Authors:** Dominique A. Cadilhac, Lauren Sheppard, Joosup Kim, Elise Tan, Lan Gao, Garveeta Sookram, Helen M. Dewey, Christopher F. Bladin, Marj Moodie

**Affiliations:** ^1^Stroke and Ageing Research Group, Department of Medicine, School of Clinical Sciences at Monash Health, Monash University, Clayton, VIC, Australia; ^2^Stroke Division, The Florey Institute of Neuroscience and Mental Health, Heidelberg, VIC, Australia; ^3^Deakin Health Economics, Institute for Health Transformation, Deakin University, Geelong, VIC, Australia; ^4^Eastern Health Clinical School, Monash University, Melbourne, VIC, Australia; ^5^Ambulance VIC, Doncaster, VIC, Australia

**Keywords:** stroke, telemedicine, economic evaluation, protocol, statistical analysis plan

## Abstract

**Introduction:** Telemedicine can address limited access to medical specialists in rural hospitals. Stroke provides an important case study because: it is a major cause of disease burden; effective treatments to reduce disability (e.g., thrombolysis) can be provided within the initial hours of stroke onset; careful selection of patients is needed by skilled doctors to minimize adverse events from thrombolysis; and there are major treatment gaps (only about half of regional hospitals in Australia provide thrombolysis for stroke). Few economic analyses have been undertaken on telestroke and the majority have been simulation models. The aim of this protocol and statistical analysis plan is to outline the methods for the cost-effectiveness evaluation of a large, multicentre acute stroke telemedicine program being conducted in Victoria, Australia.

**Methods:** Using a historical- and prospective-controlled design, we will compare patient-level data obtained in the 12 months prior to the Victorian Stroke Telemedicine (VST) program implementation and during the first 12 months of VST to determine the incremental difference in costs and patient outcomes at 3 and 12 months. Secondary aims include assessing the cost per additional patient receiving intravenous thrombolysis and the cost per additional patient receiving intravenous thrombolysis within 60 min. Tertiary aims include assessing the potential longer-term cost-effectiveness in the second year of the program at the hospitals to determine whether any program benefits are sustained once site coordinators are no longer employed; and modeling the potential net life-time costs and benefits from a societal perspective. Multivariable uncertainty and one-way sensitivity analyses will be performed to assess the robustness of results.

**Results:** Sixteen hospitals participated. Patient-level data collection including 12-month outcomes for the cohorts obtained in the first and second year of the program for each hospital was completed in January 2020.

**Conclusion:** The results from this real-world study with patient-level data will provide high quality evidence of the costs, health benefits and policy implications of telestroke programs, including the potential for application in other locations within Australia or other countries with similar health system delivery and financing.

## Introduction

Worldwide, stroke is a major cause of death and disability and represents a growing burden of disease (1). There are effective treatments to reduce the impact of this disease (2). Unfortunately, many people with stroke do not receive effective treatments for various reasons including lack of symptom recognition (only 35% arrive at hospital within 4.5 h of stroke onset) (3), limited specialist services and poor knowledge translation of evidence. Within Australia, it is estimated that over 50,000 strokes occur each year (4) and only about 10% of patients with ischaemic stroke receive intravenous thrombolysis (3).

Telemedicine for stroke (i.e., telestroke) is a model of care that can facilitate timely access to medical specialists in hospitals with limited resources for stroke care (5, 6). The establishment of telestroke networks in various parts of the world has enabled the delivery of enhanced stroke care (7) and is one of the fastest growing areas of telehealth (8). This is because of the availability of effective treatment for stroke and pressures to improve quality of care for patients, as well as the high cost of air and ground ambulance transfer (8). There are various models of care that can be developed and establishing if these are cost-effective when compared with the standard care alternatives is important for informing decisions to invest in this type of service. Few economic evaluations of telestroke have been undertaken, mainly in Europe and North America, and cost-effectiveness has been mainly determined on the basis of simulation modeling of a societal and hospital perspective (8). Two previous economic evaluations conducted alongside historical controlled studies of stroke telemedicine in the United States that have used patient-level data have shown evidence that telestroke is cost-effective (9, 10).

Following a pilot period at one hospital in regional Australia, the Victorian Stroke Telemedicine (VST) Program commenced in 2013 and was incrementally expanded to 16 hospitals by 2016 (11, 12). The main features included access to a virtual roster of neurologists for assistance with clinical decision making and employment of a site coordinator in each hospital to integrate the VST protocol into routine emergency department (ED) workflows according to an implementation plan (typically completed within 6 months; with a further 12-months for data collection and support) (13). The establishment of the cost-effectiveness credentials of a state-wide program for Australia, such as VST, is needed since no other states or territories had implemented a coordinated approach to telestroke at the time this study began, and none have used patient-level data to determine cost-effectiveness. This protocol describes the methods for the economic evaluation of the VST program and the statistical analysis plan.

## Aims

The primary aim of the economic evaluation will be to determine the cost per quality adjusted life year (QALYs) gained at 12 months after stroke.

The secondary aims are to determine the cost per additional patient receiving intravenous thrombolysis and the cost per additional patient receiving intravenous thrombolysis within 60 min of arrival to hospital.

Tertiary aims include assessing the potential long-term cost-effectiveness of the VST Program using data collected during the sustainability period of the study when telestroke had become usual practice. The “sustainability period” was the second year of the telestroke program at the hospitals when the site coordinators were no longer funded to provide direct support for implementing the VST Program. We will also seek to assess the influence of adopting different perspectives (hospital vs. societal) to the measurement of costs and benefits including modeling the potential net life-time impacts.

## Methods

### Design

The economic evaluation, a sub-study of the clinical effectiveness project (12), will be a pragmatic, real-world, economic evaluation of the implementation and long-term impacts of the VST Program compared to standard care. The study design of the VST Program evaluation has been detailed previously (11–13). In brief, the study has a pre-post-test design with a combination of historical- and prospective-control patient-level data to provide evidence on standard care practice, made possible through the iterative addition of hospitals over 7 years. After training and a short pilot phase to refine the clinical protocol at each hospital, the VST Program was implemented and clinicians then have the ability to consult with a virtual network of stroke specialist doctors for diagnosis and treatment recommendations for patients presenting to their emergency departments (EDs) with suspected stroke (13). Prior to the VST Program commencing, only five of the 16 hospitals were routinely providing intravenous thrombolysis for patients with ischaemic stroke. The project is led by the Florey Institute of Neuroscience and Mental Health. As part of this sub-study, a cost-description analysis of each comparator (pre-post) pathway will be undertaken and then compared to determine the incremental cost-effectiveness and cost-utility of the VST Program (see below).

### Setting

EDs of 16 public hospitals located in regional Victoria, Australia. Data were collected from 2010 until 2020.

### Study Perspective

A societal perspective with a major focus on the health sector will be used for this evaluation.

### Patient Eligibility Criteria

Adult patients with suspected stroke who present to the ED of participating hospitals for assessment and treatment were included. These patients are identified using the following international classification of diseases (ICD10) codes assigned to patients in the ED: G45.9, I61.9, I62.1, I62.9, I63.9, I64, R41.0, R47.0, R47.1, S06.00, S06.01) and for admitted cases: I61, I62.9, I63, I64, I67.8, I67.9, G45.0, G45.1, G45.2, G45.3, G45.8, G45.9 (13).

A screening log is used to record information for each cohort and identify patients eligible for additional data collection. Patients with a confirmed stroke and arrived at the ED within 4.5 h of symptom onset, and those provided a VST consultation (irrespective of other clinical characteristics), have additional data collected using a purposefully designed, standardized medical record audit tool (see Data collection) (13).

### Comparator “Standard Care” Control Cohort

All eligible patients who presented to the ED at a participating hospital during the 12-month period prior to implementation of VST.

### Intervention Cohort

All patients who were eligible for inclusion in the first 12-month (implementation period) or presented to hospital in the subsequent 12-months (*second year: referred to as the sustainability period*) after the implementation of the VST Program.

### Sample Size

The sample size for the economic evaluation is dependent on the number of patients presenting to the participating hospitals who meet the inclusion criteria. Data collected on over 1,000 patients will be available for the economic evaluation.

### Data Collection

Data are obtained via two methods to provide 12 months of pre-implementation data. An audit of patient records is conducted at each of the hospitals for the control group, but data collection was initially retrospective and limited to care provided and outcomes achieved in the hospital. Grant funding for this sub-study was obtained in 2015. Subsequently, where ethics approval was able to be obtained in advance of VST being implemented at a new hospital, we sought to obtain information from patients eligible for inclusion prospectively and conduct follow-up assessments at 3- and 12-months post stroke. Therefore, a sample of comparable long-term outcome data was obtained for part of the control group in this study.

Data collected using the standardized medical record audit tool includes patient characteristics on presentation to hospital (age, sex, stroke severity and pre-morbid function), management and treatment while in hospital (i.e., time of arrival and first brain imaging, treatment with intravenous thrombolysis), details of the telemedicine consultation (i.e., time and length of telemedicine consult) if applicable and outcomes at discharge from hospital (i.e., level of independence, adverse events while in hospital, discharge destination).

A waiver of consent is provided for the cohort whose data were collected retrospectively and an opt-out consent protocol is used for the cohort whose data are being collected prospectively in order to reduce the potential for selection bias. In the prospectively identified cohort, patients are provided with a consent form while in hospital or shortly after discharge from hospital.

Only patients with a confirmed stroke and who arrive at hospital within 4.5 h of symptom onset are eligible for a follow-up survey at 3-months and at 12-months to collect self-reported health outcome data (survival status, quality of life) and information on resource use (including readmissions to hospital, clinical visits, employment status and current residence). A modified Dillman protocol is used where the follow-up survey is posted to the patient's address 1 week prior to the assessment time point (14). If no response is received within 4 weeks, a second copy of the survey is sent to the patient's address and the address of a nominated alternate contact. If no response is received within 6 weeks of the initial contact attempt, program staff attempt contact with the patient and nominated alternate contact via telephone to complete the survey questionnaires. Patients are considered to be “lost to follow-up” if contact is unable to be made by 6 months post-stroke for the 3-month follow-up or by 15 months post-stroke for the 12-month follow-up survey.

### Identification, Measurement, and Valuation of Benefits

For QALY estimations, self-reported health-related quality of life (HRQoL) data were collected from patients or their proxy at 3- and 12-months using the EQ-5D-3L questionnaire (15, 16). The most appropriate published algorithm to convert the responses into utility values for an Australian population will be used (17). The Assessment of Quality of Life tool (AQoL-4D) (18), which has been validated in an Australian population of stroke survivors (19), was also collected in order to provide alternate utility values for sensitivity analyses.

Functional status was assessed using the modified Rankin scale (mRS) (20, 21). This was collected as a measure of the patient's functional status before stroke (obtained from health status information recorded at time of admission), at discharge (days 7–10, or on day of discharge if before day 7), as well as at 3- and 12-months follow-up for both the intervention and prospective control comparator groups. Due to the inherent difficulties of administering the HRQoL instruments to new stroke patients, the mRS at Day 0 will be used as a surrogate measure of patient utility in replacement of EQ-5D during the acute phase. This is done to allow the change in quality of life from baseline to 3 and 12 months to be reasonably estimated.

Additionally, the Australian Stroke Clinical Registry (AuSCR) will be used to supplement the clinical or follow-up data for patients admitted with stroke (regardless of intervention), as appropriate (22, 23). Data that can be obtained from AuSCR include patient admission details, acute clinical data, discharge information and HRQoL (using the EQ5D-3L and the mRS) and data on deaths from linkage with the National Death Index. Hospitals participating in the VST program are required to commence data collection for AuSCR by the time they complete the implementation stage to permit the sustainability phase data to be obtained through routine data collection processes (13).

### Identification, Measurement, and Valuation of Costs

The costs included in this analysis comprise intervention costs, direct healthcare, productivity losses/gains and relevant patient out-of-pocket costs. Costs will be valued based on the reference year 2018, and a discount rate of 3% will be applied (24). Table 1 includes a detailed list of resource use items and costs collected and their sources.

**Table 1:** Resource use information collected and sources of unit prices.

**Cost category**	**Unit**	**Data collection method**	**Person responsible for data collection**	**Potential source of unit cost information**
**Acute**				
Telemedicine neurologist time	Minutes	Case Report Form/Neurologist Case Report Form	Site Coordinator/Telemedicine neurologist	Paid invoice, average cost
Diagnostic tests undertaken (regardless of destination)	Type of test	Case Report Form	Site Coordinator	Medicare Benefits Schedule
rt-PA treatment	Dose	Case Report Form	Site Coordinator	Industry
Ambulance transfers (regardless of destination)	Yes/No	Case Report Form	Site Coordinator	Ambulance Victoria
Acute inpatient stay	Bed days	Case Report Form	Site Coordinator	IHPA, National Hospital Cost Data Collection.
**Post-acute**				
Inpatient rehabilitation program	Bed days	Case Report Form	VST Program officers/site coordinator	IHPA, National Hospital Cost Data Collection.
Outpatient rehabilitation services/stroke-related therapy	Type of service, no. of times provided	Follow-up questionnaire	VST Program officers/ site coordinator	Comcare/Medicare Benefits Schedule
Hostel/Nursing home	Length of stay	Follow-up questionnaire	VST Program officers/site coordinator	Aged Care Financing Authority,
Transitional Care	Length of stay	Follow-up questionnaire	VST Program officers/site coordinator	Aged Care Financing Authority
Other hospital re-admissions	Bed days	Follow-up questionnaire	VST Program officers/site coordinator	IHPA, National Hospital Cost Data Collection.
Hospital admission for recurrent stroke recurrence	Yes/No	Follow-up questionnaire	VST Program officers/site coordinator	IHPA, National Hospital Cost Data Collection.
Home modifications	Type of modification	Follow-up questionnaire	VST Program officers/site coordinator	Online search of suppliers
Special equipment and aids	Type of equipment	Follow-up questionnaire	VST Program officers/site coordinator	Online search of suppliers
Medications	Type of medication	Follow-up questionnaires	VST Program officers/site coordinator	Department of Health, Schedule of Pharmaceutical Benefits
**Patient/caregiver**				
Changes in accommodation	Type of accommodation, length of stay	Follow-up questionnaires	VST Program officers/site coordinator	Aged Care Financing Authority
Home modifications	Type of modification	Follow-up questionnaires	VST Program officers/site coordinator	Specified by patient
Special equipment and aids	Type of equipment	Follow-up questionnaires	VST Program officers/site coordinator	Specified by patient
Medications	Type of medication	Follow-up questionnaires	VST Program officers/site coordinator	Department of Health, Schedule of Pharmaceutical Benefits
Informal care	Hours	Follow-up questionnaires	VST Program officers/site coordinator	Fair Work Commission
Outpatient rehabilitation services/stroke-related therapy	Type of service, no. of times provided	Follow-up questionnaires	VST Program officers/site coordinator	Specified by patient
**Societal**				
Changes in workforce participation	Full time/part time/not-working	Follow-up questionnaires	Site coordinator	Australian Bureau of Statistics, average Australian adult wage
**Intervention**				
Cost of maintaining 1,800 line	Usage fee and monthly charge	Program management	VST Program staff at Florey	Paid invoice
Neurologist on-call	Standard on-call rate	Program management	VST Program staff at Florey	Paid invoice
Software upgrades/licensing	Yes/No	Program management	VST Program staff at Florey	Paid invoice
Capital equipment	Yes/No	Program management	VST Program staff at Florey	Paid invoice
Administrative overheads: - Site Coordinator time - Education and training time	EFT Participant time, venue and material costs	Program Management Site Coordinator Education & Training diary	VST Program staff at Florey/site coordinator Site coordinator	Hourly rate Paid invoice

*VAED, Victorian Admitted Episodes Data Set; HACC, Home and Community Care; ABS, Australian Bureau of Statistics; VST, Victorian Stroke Telemedicine; EFT, Equivalent full-time, IHPA, Independent Hospital Pricing Authority*.

Several resource use items relevant to economic evaluations of interventions for stroke (25) are not being collected in order to reduce responder burden and because they are unlikely to have been influenced by the VST Program; these include medical visits in the community, use of medications that are not secondary prevention medication, respite care, volunteer time, and caregiver impacts.

#### Direct Costs of Delivering the Stroke Telemedicine Program

Intervention-related expenses collected via program management processes include the cost for administrative staff including office space, the toll-free telephone line, on-call fees to neurologists, software and licensing costs for the telestroke service, and capital equipment expenses. Local site coordinators were only employed during the set up (~6-months) and implementation (12 month) phases at the hospitals. Their role was to support the clinical implementation and collect data for the research. Only the clinical implementation costs of local site coordinators will be counted as part of the intervention-related expenses. In the second year, these roles were not funded and will not be included in the program delivery (intervention-related) expenses.

#### Direct Healthcare Costs of Participants

Resource use during the acute phase is captured via auditing of patient medical records. Neurologist consultation time via telestroke is captured through the Neurologist Consultation Form. To estimate the hospital costs, we will access published national hospital cost data (Table 1). The national efficient price for the stroke/TIA diagnostic-related group (DRG) categories will be mapped to each patient based on diagnosis, severity on presentation, length of stay and discharge destination.

Follow-up questionnaires were administered to the intervention and prospective control groups at 3- and 12-months post discharge to collect resource use information and other costs incurred during the post-acute hospital phase. Participants were asked to provide information on changes in accommodation, hospital re-admissions, stroke recurrence, and other ongoing stroke-related resource use such as outpatient rehabilitation services, medications, home modifications, equipment and aids purchased.

#### Productivity and Informal Care (Indirect) Costs

Information on informal care and changes to employment were collected at 3- and 12-months post discharge to estimate indirect costs. Productivity losses or gains of patients will be calculated based on the average earnings by age and gender available from the Australia Bureau of Statistics (26). Hours of informal care will be valued using published average unit costs of formal caregivers.

### Data Analysis

The data analyses will be performed using Microsoft Office Excel or STATA software (StataCorp. 2019. State Statistical Software: Release 16. College Station, TX: StataCorp LLC), with add on software as required for probabilistic multivariable analysis. Descriptive statistics, using parametric and non-parametric methods appropriate for the data will be used to summarize the pooled data for the control (pre-implementation) and intervention cohorts. Multivariable or multilevel models (with level defined as hospitals) will also be undertaken. For example, generalized linear regression modeling of costs with gamma distributions and log linked for multivariable analyses that adjust for age, gender, stroke-type, stroke severity, length of stay and patient clustering by hospital as part of the cost-description analyses may be appropriate to account for the non-parametric cost data. The results will then be used as inputs in the incremental cost-effectiveness and cost-utility analyses.

Multiple imputation methods and population weighted adjustment methods will be applied to address the missing data for variables that are found to have a large impact on the healthcare services costs (27).

#### Sensitivity Analyses

Deterministic and probabilistic sensitivity analysis will be performed to determine the level of confidence around the resulting incremental cost-effectiveness ratios (ICERs). Deterministic (one-way) sensitivity analysis will be conducted by varying the key parameters (such as unit cost of resource use, quantity of resource use, mRS outcome by cohort, QALYs) in the with-in trial economic evaluation. Responses to the Assessment of Quality of Life questionnaire (18) and utilities based on mRS may also be used as alternate sources for estimating QALYs. Results from the probabilistic sensitivity analyses will be presented in tornado diagrams in order to compare the relative importance of the parameters to the ICER. Bootstrapping analysis will be conducted to examine the robustness of the result by simulating a minimum of 2,000 iterations for the incremental cost and QALY regardless of significance in the between-group difference. The results from the probabilistic sensitivity analysis will also be presented using a cost effectiveness acceptability curve (CEAC). The CEAC indicates the probability that the intervention is cost-effective compared with usual care, based on the commonly used willing-to pay threshold of AUD50,000 per QALY gained (28, 29).

#### Long-Term Modeling

Since patient level data as part of this study have been collected up to 12-months post stroke, any estimated costs and outcomes in the longer-term will need to be modeled. A Markov model will be built in TreeAge Pro 2019, R2 (Treeage Software Inc., Williamston, Massachusetts, USA) to estimate the costs and effects of the VST program on the cohort, compared to the usual care group over its lifetime. This will account for seven health states associated with stroke (defined by mRS scores) to simulate the costs and benefits based on a life time horizon. Health benefits gained in the long term may translate into cost savings to offset implementation/intervention costs.

## Discussion

Telestroke has the potential to improve access to best-practice stroke care and reduce mortality (13, 30). This protocol and statistical analysis plan provides an outline of the methods for the economic evaluation of the VST Program. Most of the previous economic evaluations provide support for telestroke being a cost-effective or cost saving strategy. However, only two trial-based economic evaluations have been conducted, the Providence Oregon Telestroke Network and the Access to Critical Cerebral Emergency Support Services (9, 10). It is worth nothing that all previous evaluations have found improvement in health benefits for patients within a telestroke network compared to usual care (9, 10, 31–35). One cost utility analysis found telestroke to be cost saving within as little as 90 days, mainly due to the reduction of inter-hospital transfers (10). On the other hand, other evaluations determined telestroke to be not cost-effective in the short term (32, 34). However, when longer time horizons were adopted, the cost-effectiveness of telestroke consistently improved over time. This was attributable to larger upfront implementation costs that were offset by greater health benefits and savings achieved in the longer term (such as decreased nursing home care required).

There are also limitations to the study since it is a “pre-post” design and may lack internal validity and be open to various forms of bias including reporting and selection bias. However, designing a blinded randomized controlled trial was impractical and unethical for the VST Program. Further, as this is a staggered implementation where patients were recruited at different hospitals between 2010 and 2017, there were adaptations to the health system over time. The follow-up resource use questionnaires are also subject to recall bias. Lastly, for the comparator group, there is no follow-up information for the historical cohort beyond the hospital setting and the prospective data could only be obtained from relatively few control patients at 3-months.

The strengths of our methods for this economic evaluation include the reliable patient-level data in the acute phase and detailed intervention costs. The final sample sizes for the clinical effectiveness study as of 31 December 2017 were 2,887 patients with suspected stroke in the control period and 3,178 patients with suspected stroke in the VST intervention period. There were no major differences in patient characteristics with suspected stroke between study periods, however more were provided an initial diagnosis of ischaemic stroke and had a documented time from symptom onset in the VST Program implementation cohort (13). The number of eligible patients for detailed audit was 423 for the control period and 601 in the intervention period (13). As of January 2019, 360 of the 764 eligible patients who could be sent an outcome survey at 3-months have provided survey responses following hospital admission (26 patients in the control period and 334 patients in the intervention period). Information from another 101 patients have been collected at 12 months after their hospital admission (88 in the control period and 13 in the intervention period).

Another strength of the economic evaluation will be the use of the EQ-5D to calculate the utility score for estimating QALYs gained. This will allow VST results to be compared with international studies which often use the EQ-5D instrument. In a recent review, there were very few well designed economic evaluations that could be used to usefully inform public health policy and investment decisions in general, highlighting an urgent need for greater application of economic evaluation to understand the cost-effectiveness of alternative implementation efforts (36). Our work will be an important contribution to the field of stroke and implementation research that is focused on improving uptake of evidence-based interventions in hospital settings.

The results of this economic evaluation will inform the decision to develop stroke telemedicine services in other states within Australia. Countries with similar financing and health system models of care may find this information relevant for policy and planning. If deemed cost-effective, the evidence from this study will support expanding telestroke services for improving stroke care and reducing inequities between regional/rural areas and metropolitan locations. Evidence of the sustainability of telestroke services as an effective model for improving the quality of care in the treatment of acute stroke is urgently needed.

## Ethics Statement

The studies involving human participants were reviewed and approved by Human research ethics committee (HREC) approval was provided by Monash University reference number: 2014-2482-2291 and Deakin University reference number: 2014-297. All hospitals contributing patient data for the Victorian Stroke Telemedicine project also provided local ethics and governance approvals with Bendigo Health Care Group Human Research Ethics Committee as the lead HREC. The patients/participants provided their written informed consent to participate in this study where relevant.

## Author Contributions

DC: conceived the study and co-led the VST Program, obtained the grant funding, designed the study, and drafted the manuscript. LS: designed the study and revised the manuscript. JK: coordinated the collection of resource use data at follow-up, designed the study, and drafted the manuscript. ET and LG: contributed to aspects of the study design and drafts of the manuscript. GS: contributed to revisions of the manuscript. HD: obtained grant funding, contributed to design of the study, and revised the manuscript. CB: co-led the VST Program, obtained the grant funding contributed to design of the study, and revised the manuscript. MM: obtained grant funding, contributed to the study design, and drafted the manuscript. All authors agree to be accountable for the content of the work.

## Conflict of Interest

DC and CB declare grants from Boehringer Ingelheim paid to their institution in support of the Victorian Stroke Telemedicine project. DC declares grants from Boehringer Ingelheim, Medtronic, Shire, Pfizer, Amgen, Bristol-Myers Squibb outside the submitted work. The remaining authors declare that the research was conducted in the absence of any commercial or financial relationships that could be construed as a potential conflict of interest
